# Supplementary material for: Application of Pulsed Electric Fields to Pilot and Industrial Scale Virgin Olive Oil Extraction: Impact on Organoleptic and Functional Quality
Source: Foods. 2022 Jul 8;11(14):2022. doi: 10.3390/foods11142022 (PMC9318511; doi:10.3390/foods11142022)
Supplement: Supplementary file 1 [file foods-11-02022-s001.zip › foods-1789003-supplementary.pdf]

Table S1: Volatile composition of control and PEF oils obtained from 'Manzanilla' and 'Hojiblanca' cvs

		First pilot trials	
volatile content (ng g^{-1} oil)		Manz control	Manz PEF
aldehydes C6/LnA	(*E*)-hex-3-enal	33,3±2,0	33,8 ±4,3
	(*Z*)-hex-3-enal	1026,2±157,7	1372,2±77,3
	(*Z*)-hex-2-enal	329,3±6,1	388,5±44,6
	(*E*)-hex-2-enal	11971,33±501,2	13218,6±44,3
alcohols C6/LnA	(*E*)-hex-3-enol	7,2±0,2	9,2 ±5,7
	(*Z*)-hex-3-enol	1390,8±4,5	1357,1±7,4
	(*E*)-hex-2-enol	252,2 ±1,1	216,1±20,2
aldehydes C6/LA	hexanal	415,2±0,8	507,4±17,5
alcohols C6/LA	hexan-1-ol	999,0**a**±11,6	1044,7**b**±8,1
carbonyls C5/LnA	pent-1-en-3-one	220,1±8,7	242,3±2,7
	Z2-pentenal	81,8±4,0	77,4±0,5
	E2-pentenal	20,6±10,7	70,0±76,0
alcohols C5/LnA	1-penten-3-ol	260,6±18,1	226,7±38,0
	(*Z*)-pent-2-enal	1417,7±17,3	1483,1±67,9
	(*E*)-pent-2-enal	792,1±22,3	808,1±137,3
carbonyls C5/LA	pentan-3-one	40,8±0,9	50,8±21,2
	pentanal	202,3±9,4	176,0±43,8
alcohols C5/LA	pentanol	36,6±13,2	46,4±0,9
Penten dimers	DP-1	191,3±1,1	241,6±7,68
	DP-2	167,3**a**±0,8	213,1**b**±4,2
	DP-3	889,8**a**±19,7	1261,1**b**±33,4
	DP-4	82,7±12,0	79,2±15,6
	DP-5	448,5±6,4	666,7±55,03
	DP-6	818,53±9,0	878,0±23,9
	DP-7	109,8±3,7	111,4±15,2
LOX Esters	hexyl acetate	1042,3±15,9	1094,0±114,2
	(*E*)-hex-2-en-1-yl acetate	33,7±1,7	42,2±5,8
	(*Z*)-hex-3-en-1-yl acetate	207,3±3,5	214,1±19,1
non- LOX esters	Methyl acetate	7,2±1,9	11,5±8,0
	Ethyl acetate	5,0±0,8	4,5±1,0
	Methyl hexanoate	46,4 ±31,9	32,44±7,3
	Ethyl hexanoate	378,4±247,2	303,3±60,3
aldehydes AA	2-methyl-butanal	276,4±1,3	287,1±36,1
	3-methyl-butanal	154,9±7,1	142,3±20,6
alcohols AA	2-methyl-butan-1-ol	5,4±2,4	9,4±2,9
terpenes	limonene	69,2±89,5	23,1±18,8
	ocimene	9269,9±117,9	9422,7±1715,3

Table S1 (cont)

volatile content (ng g^{-1} oil)		Second pilot trials<br>Manz control	Manz PEF
aldehydes C6/LnA	(*E*)-hex-3-enal	42,58±2,2	45,9±1,9
	(*Z*)-hex-3-enal	2527,8±553,4	2958,4±515,5
	(*Z*)-hex-2-enal	285,2±1,2	295,5±19,0
	(*E*)-hex-2-enal	16204,4±744,7	14664,1±582,4
alcohols C6/LnA	(*E*)-hex-3-enol	9,3±2,7	6,12±0,67
	(*Z*)-hex-3-enol	1991,5±141,4	2137,7±143,5
	(*E*)-hex-2-enol	193,4±2,9	177,4±8,1
aldehydes C6/LA	hexanal	426,4±0,0	424,8±4,3
alcohols C6/LA	hexan-1-ol	1272,8±94,9	1398,7±93,8
carbonyls C5/LnA	pent-1-en-3-one	136,9±9,3	139,2±7,59
	Z2-pentenal	50,0±0,7	50,76±0,9
	E2-pentenal	20,0±7,2	18,9±6,0
alcohols C5/LnA	1-penten-3-ol	167,8±7,8	152,0±4,7
	(*Z*)-pent-2-enal	2381,0±238,8	2615,7±243,2
	(*E*)-pent-2-enal	461,7±66,4	475,4±62,7
carbonyls C5/LA	pentan-3-one	11,5±0,6	13,4±4,6
	pentanal	82,37**a**±0,5	86,0**b**±0,8
alcohols C5/LA	pentanol	34,7±22,7	30,9±8,7
Penten dimers	DP-1	114,3±1,3	115,4±5,7
	DP-2	104,0±5,3	100,8±7,9
	DP-3	588,7±42,7	587,2±44,6
	DP-4	22,4±9,3	61,6±0,5
	DP-5	398,10±77,9	324,9±28,0
	DP-6	667,6±28,47	653,5±2,5
	DP-7	129,3±10,0	124,8±57,1
LOX Esters	hexyl acetate	2014,8±186,8	2153,4±191,1
	(*E*)-hex-2-en-1-yl acetate	37,9±1,52	39,0±2,9
	(*Z*)-hex-3-en-1-yl acetate	187,2±21,9	195,3±19,8
non- LOX esters	Methyl acetate	7,6±0,3	8,5±0,4
	Ethyl acetate	1,6±0,1	1,6±0,1
	Methyl hexanoate	21,2±4,5	20,5±0,7
	Ethyl hexanoate	369,34±330,67	190,8±28,2
aldehydes AA	2-methyl-butanal	40,4±4,9	38,2±4,0
	3-methyl-butanal	15,77±2,34	14,0±1,7
alcohols AA	2-methyl-butan-1-ol	7,6±7,8	25,4±23,4
terpenes	limonene	58,4±18,5	71,1±2,9
	ocimene	9380,9±1387,1	9672,9±1202,2

Table S1 (cont)

volatile content (ng g-1 oil)		Third pilot trials<br>Hojib control	Hojib PEF
aldehydes C6/LnA	(*E*)-hex-3-enal	67,0±9,1	77,8±0,0
	(*Z*)-hex-3-enal	5600,0±295,1	6144,8±0,0
	(*Z*)-hex-2-enal	457,04**a**±125,4	595,6**b**±0,0
	(*E*)-hex-2-enal	2678,3±2,3	3358,42±0,0
alcohols C6/LnA	(*E*)-hex-3-enol	16,5±11,6	9,9±0,0
	(*Z*)-hex-3-enol	4009,2±74,7	3964,6±0,0
	(*E*)-hex-2-enol	252,6±10,4	243,8±0,0
aldehydes C6/LA	hexanal	1389,0**a**±18,7	1595,8**b**±0,0
alcohols C6/LA	hexan-1-ol	3926,3±63,7	3921,4±0,0
carbonyls C5/LnA	pent-1-en-3-one	87,3±0,9	98,7±0,0
	Z2-pentenal	71,0**a**±2,1	79,0**b**±0,0
	E2-pentenal	21,8±5,3	20,4±0,0
alcohols C5/LnA	1-penten-3-ol	46,9±12,7	65,5±0,0
	(*Z*)-pent-2-enal	2438,7±7,7	2356,7±0,0
	(*E*)-pent-2-enal	234,1±11,9	222,2±0,0
carbonyls C5/LA	pentan-3-one	7,2**a**±0,1	9,2**b**±0,0
	pentanal	61,1±3,1	62,6±0,0
alcohols C5/LA	pentanol	40,7±12,6	52,0±0,0
Penten dimers	DP-1	71,9±5,5	99,3±0,0
	DP-2	60,2±2,5	79,22±0,0
	DP-3	955,6±25,5	991,±0,0
	DP-4	23,6±1,6	27,8±0,0
	DP-5	209,4**a**±0,5	237,1**b**±0,0
	DP-6	829,7±460,1	777,4±0,0
	DP-7	1233,6±40,2	1542,9±0,0
LOX Esters	hexyl acetate	2985,4±11,6	2982,4±0,0
	(*E*)-hex-2-en-1-yl	28,7±4,5	19,2±0,0
	(*Z*)-hex-3-en-1-yl	46,9±1,9	42,4±0,0
non- LOX esters	Methyl acetate	7,5±0,3	8,4±0,0
	Ethyl acetate	1,9±0,5	1,5±0,0
	Methyl	42,9±26,4	73,5±0,0
	Ethyl hexanoate	83,9±40,7	125,9±0,0
aldehydes AA	2-methyl-butanal	25,9±1,2	27,3±0,0
	3-methyl-butanal	12,1±2,1	11,9±0,0
alcohols AA	2-methyl-butan-1-	22,7±0,6	28,1±0,0
terpenes	limonene	58,7**a**±37,4	81,9**b**±0,0
	ocimene	2563,4±120,7	2684,3±0,0

Table S1 (cont)

volatile content (ng g⁻¹ oil)		Industrial trials: Manz control	Industrial trials: Manz PEF
aldehydes C6/LnA	(*E*)-hex-3-enal	23.7±4.9	20,1±4,2
	(*Z*)-hex-3-enal	1691.1±19.8	1803±62,3
	(*Z*)-hex-2-enal	199.1±28.3	220,6±10,9
	(*E*)-hex-2-enal	1762.2±147.9	2067,8±191,3
alcohols C6/LnA	(*E*)-hex-3-enol	15.1±11.1	3,9±1,6
	(*Z*)-hex-3-enol	1643.0±137.4	1636,2±152,7
	(*E*)-hex-2-enol	143.7±9.9	131,4±11,3
aldehydes C6/LA	hexanal	386.4±21.4	460,6±55,1
alcohols C6/LA	hexan-1-ol	674.1±63.3	651,3±63,6
carbonyls C5/LnA	pent-1-en-3-one	101.0±12.2	103,9±14
	Z2-pentenal	11.8±1.0	11,4±0,9
	E2-pentenal	15.1±1.4	16±3
alcohols C5/LnA	1-penten-3-ol	44.3±3.7	47,4±6,2
	(*Z*)-pent-2-enal	452.5±49.0	427,6±69,4
	(*E*)-pent-2-enal	606.9±104.9	611,2±105,6
carbonyls C5/LA	pentan-3-one	19.5±0.2	22,1±0,2
	pentanal	55.6±6.1	57,7±9,7
alcohols C5/LA	pentanol	8.8±0.6	8,9±2,2
Penten dimers	DP-1	76.4±14.6	81,5±18,2
	DP-2	93.1±6.0	95,8±16
	DP-3	623.0±99.6	665,5±113,2
	DP-4	5.5±2.0	6,1±2
	DP-5	421.1±24.9	477,9±43,9
	DP-6	509.9±89.1	514,8±74,3
	DP-7	154.9±70.3	86,6±34,4
LOX Esters	hexyl acetate	878.4±71.1	876,4±58,6
	(*E*)-hex-2-en-1-yl acetate	49.4±1.3	45,1±0,3
	(*Z*)-hex-3-en-1-yl acetate	3021.8±320.2	2805±211
non- LOX esters	Methyl acetate	5.1±1.7	4,5±1,5
	Ethyl acetate	2.3±0.3	2,2±0,6
	Methyl hexanoate	15.8±2.5	18,1±5,9
	Ethyl hexanoate	8.2±1.0	7,7±0,7
aldehydes AA	2-methyl-butanal	17.6±1.2	19,7±2,9
	3-methyl-butanal	6.1±0.5	6,1±0,7
alcohols AA	2-methyl-butan-1-ol	0.3±0.4	1,1±0,6
terpenes	limonene	7.2±1.8	5±2,8
	ocimene	10024.5±2802.3	9751,5±2564,7
